# Correction: Framing COVID-19: How we conceptualize and discuss the pandemic on Twitter

**DOI:** 10.1371/journal.pone.0306644

**Published:** 2024-07-02

**Authors:** Philipp Wicke, Marianna M. Bolognesi

In the Identifying topics in Covid-19 discourse on Twitter through topic modeling subsection of the Study design, a reference is omitted from the first sentence of the second paragraph.

The correct sentence is: Conversely, in communication sciences a frame is typically defined as consisting of two elements [28]: elements in a text such as words, used as framing devices, and (latent) information used as reasoning devices, through which a problem, cause, evaluation, and/or treatment is implied. (Burgers et al., 2016).

The reference is: Burgers, C., Konijn, E.A. and Steen, G.J. (2016), Figurative Framing: Shaping Public Discourse Through Metaphor, Hyperbole, and Irony. Commun Theor, 26: 410–430. doi:10.1111/comt.12096

The following information is missing from the acknowledgement section: We are also thankful to Christian Burgers and Johannes Wiesner for providing advice on part of the statistical tests that we adopted in the study.
