# Supplementary material for: Framing COVID-19: How we conceptualize and discuss the pandemic on Twitter
Source: PLoS One. 2020 Sep 30;15(9):e0240010. doi: 10.1371/journal.pone.0240010 (PMC7526906; doi:10.1371/journal.pone.0240010)
Supplement: Attachment — Submitted filename: Reviewer 5.docx [file pone.0240010.s001.docx]

Reviewer 5

General comments:

This article provides some interesting first insights into what topics are discussed in relation to the Covid-19 pandemic on Twitter and what metaphor frames are prevalent. It was slightly disappointing that the research questions are purely descriptive, but maybe that is inevitable for a study that must be one of the first on the topic.

What is headed 'theory section' in fact  includes both a literature review on analysing tweets on epidemics and another part on metaphor theory, but the two parts are not linked well. Also, the results are not linked back to the studies featuring in the literature review.

The paper seems to have been written quickly and was clearly not proofread.

Specific comments:

- As for the corpus, how can the authors be sure that the corpus only contains tweets by non-experts?
- For Study 2, why did the authors decide against lemmatisation? There are good reasons not to use that process, e.g. because different forms of a lemma can express different metaphor scenarios, but the reasons should be spelled out.
- The #ReframeCovid also includes storm, monster and tsunami source domains, and it does not only include examples from news media.
- What are possible labels for the 16 topics?
- Why was family chosen as a literal control frame?
- "Previous literature shows that metaphor-related words cover only a percentage of the discourse": does this refer to Steen et al. (2010)?
