# Supplementary material for: Framing COVID-19: How we conceptualize and discuss the pandemic on Twitter
Source: PLoS One. 2020 Sep 30;15(9):e0240010. doi: 10.1371/journal.pone.0240010 (PMC7526906; doi:10.1371/journal.pone.0240010)
Supplement: Attachment — Submitted filename: Response to Reviewers.docx [file pone.0240010.s002.docx]

Reply to reviewers

**Framing COVID-19 How we conceptualize and discuss the pandemic on Twitter** PONE-D-20-10986.

We are very thankful to all 5 reviewers for the constructive feedback and comments, which helped us improve our manuscript in many ways. We took onboard all comments and we hereby provide our reply to each and every point raised. We apologize for the length of this document, which matches the length of the actual article.

A note to all reviewers: The matching algorithm used to identify the target terms in the corpus has been improved (notably, it is not case-sensitive anymore, so it can retrieve for example occurrences of “fighting” as well as “FiGHting”: this is quite relevant because on social media capital letters and mixes of small and caps are often used). We have therefore updated the numbers in the “Framing results” with more accurate values (none of which are affecting the topic modeling, interpretation or discussion). Accordingly, Fig. 6 and Table 2 were updated as well.

**Reviewer #1:**

*The paper analyzes Tweets that are used to talk about Covid-19 in three studies. The first identifies topics of these tweets. The second quantifies the prevalence of WAR metaphors in general, and across the topics identified in the first study. The third explores alternative figurative frames for the virus, along with one non-figurative frame (FAMILY). It is a timely study with interesting methods and results. I enjoyed reading it. I have suggestions for a revision, along with some methodological questions detailed below.*

Reply: Thank you.

*Introduction

1. The introduction could be better connected with the methods used in and research questions explored in the studies. Why is it interesting and important to determine what topics are discussed on Twitter in relation to Covid-19 or how frequently WAR and other metaphors are used? For example, could the topic modeling help to improve surveillance and forecasting about the disease? What frequency of WAR metaphors should we expect? How and why were the specific alternative frames chosen? Are there relevant theories in cognitive linguistics that the current work informs?*

Reply: Thank you for this comment. The introduction contains the following information: topic (first paragraph), how we operationalize the topic (through tweets, 2nd paragraph), aim (3rd para) and RQs (4th para). The information required by the reviewer in our opinion does not belong in the introduction, but rather in the Theoretical background, which contains related work in cognitive linguistics, and Methods, where we explain why specific frames were used etc. Moreover, we have already included in the Introduction that we expect to find WAR related metaphors (indicating a very specific expected frequency of metaphors to be found appears to be out of place). We took this comment onboard by adding a very short paragraph at the end of the introduction in which we highlight the relevance of this study, which is then developed in the next section.

*2. The emphasis and major strength of the paper seems to be the focus on WAR metaphors. The prevalence of WAR metaphors is quantified for an important issue, by topic, and in the context of other metaphoric frames (and a non-metaphoric frame). To highlight these strengths, I would encourage the authors to emphasize the novelty of quantifying the WAR frame using automated methods and real world data. Maybe switching the order of studies 1 and 2 would be helpful? (The results of Study 1 are difficult to interpret on their own).*

Reply: Thank you. We emphasized these strengths in the last paragraph of the introduction. Moreover, following also the suggestion by reviewer 2, we have restructured the paper (methods and analyses), and thus the three studies are now three parts of the same study, so they don’t have to be interpreted by their own. This should improve the readability and solve this comment too.

*3. The “Theoretical Background” section describes past work that is certainly interesting and relevant, but it doesn’t really identify theoretically motivated questions that the current work is well-suited to address. In most cases, it emphasizes practical applications and real world issues and/or methods (e.g., the relationship between measuring public sentiment on Twitter and addressing public health issues). Maybe rename this section for clarity.*

Reply: Thank you. The Theoretical Background section contains related works (theoretical background) in the following fields, which we bring together in our study: quantitative analyses based on Twitter data related to epidemics (paragraphs 1-4); cognitive linguistic studies of figurative framings and metaphors, including the WAR frame and metaphors that we tackle in our study (paragraphs 5-7) and alternative framings (paragraph 8). This section does contain a selected review of the literature in these two fields of research, which is functional to our argumentation. We therefore stick to the label Theoretical Background.

*Smaller points related to the introduction:
4. On p. 3 the authors note that around “16K Tweets are posted by Twitter users every hour, containing a hashtag such as #coronavirus, #Covid-19 or #COVID.” Is this based on the current data collection or a metric that is computed by Twitter? What is the temporal window for this statistic?*

Reply: Thank you. We have made this relation clearer by stating that “collecting every tweet containing a hashtag such as #coronavirus, #Covid-19 or #Covid, we accumulated around 16,000 tweets within an hour each day.” Meaning, that the metric is in fact based on the current data collection. This ambiguity has now hopefully been resolved.

*5. It seems like there is a typo on p. 4: “Unlike the articles on magazines and journals typically used for corpus analyses of this kind, Twitter does contain messages written by journalists and other experts in mass communication, as most tweets are provided by non-expert communicators.” Maybe “as” should be “although”?*

Reply: Thank you. We solved this.

*Methods
1. Please clarify the following questions:
a. How were the 25k tweets per day selected? It seems like the algorithm picked up the first 25k tweets that included one of the specified hashtags per day, but that’s not explicitly stated. How long did it typically take to get to 25k tweets?
b. Were retweets included?
c. For a given person who tweeted multiple times in a day about the virus, was it only their first tweet that was included? What was the filtering criteria/method?
d. Were Tweets in languages other than English included?*

Reply: 1a) The algorithm sends a query to the Twitter API. Our query asks for 25,000 tweets from one day that contain a list of specified hashtags (#CoronaVirus, #Covid etc.) and are of English language. Twitter restricts the access by firstly not allowing to choose a time of day in the query, therefore we receive the first 25k tweets on that day. Secondly, there is a rate limit to retrieve tweets. To abide by that rate limit, the algorithm has waiting periods. On average it takes about 2hrs and 45min to collect 25k tweets from one day.

Reply:	1b) Retweets were not included (as we explained in the paper).

Reply:	1c) Yes, only the first tweet about the pandemic was retained, for each user. In this way we constructed a balanced corpus that had one tweet per user. By doing so, we avoided having, for example, hundreds of tweets from the same user who, maybe, was particularly keen on using the WAR frame. This would have biased our results. Instead, by keeping 1 tweet per user we have a better overview of how people talk about covid on twitter. The algorithm picked the first tweet per user that it could find, within the timeframe indicated. Collecting all tweets from all users, and then randomly selecting one per each user would have been computationally cumbersome and would have exceeded our database capacity.

Reply: 1d) see a), we have only selected English tweets through Twitter's API language filter.

*2. It should be possible to clarify some of the hedging in the sentence, “our corpus arguably encompasses mainly tweets produced by users residing in the USA, where the time of data collection corresponds to awake hours, and the targeted language corresponds to the first language of many (if not most) US residents” (p. 12). I realize that the location data was stripped from the text before it was stored, but the text is initially tagged with the location data, so maybe it is possible to estimate the percentage of tweets from the US. Language use questions are asked on the US census every 10 years and roughly 80% of US residents report speaking English at home. A citation would make the case stronger.*

Reply: In fact, a preliminary analysis of tweets included a statistical analysis of the location data. To the best of our knowledge, the location data provided by the free Twitter API is self-reported by the user. Unfortunately, as the data included a great range of responses from “*Anywhere that has liquor*” or “*Here.*” to “*México!!!*” or “*Universe*”, we found no feasible way as to manually interpret the sample. We concluded that most of the locations indicated the US. Furthermore, the tweet time and targeted language seemed to be a more reliable estimator to conclude the majority of tweets being from US citizens. We have now added a citation, supporting the claim that most US residents speak English at home.

*3. How were the topic numbers (n = 4 vs. 16) decided in the first study? I find the results of this study hard to interpret. I think they are more informative when presented in concert with the results of study 2.*

Reply: Thank you. We have added a sentence explaining that the numbers 4 and 16 were chosen on an empirical basis and then backed up by an analysis of the internal coherence of the clusters. We were interested in a small but meaningful number of clusters and opted for 4, because 1-2-3 were in our opinion too limited; then we were also interested in a much more granular solution, so we opted for 16 (4 x 4).

Reply: We have now reported the coherence scores for these clusters for different LDA inference algorithms (as suggested by Reviewer 4). A more detailed discussion of these scores, which is linked to the decision to choose topics 4 and 16 is given in response to Reviewer 4. In short, the methods used to evaluate the internal coherence of the various cluster solutions from 1 cluster (1 topic) to 20 clusters (20 topics), shows that the coherence is particularly good for 4 clusters (according to the elbow method of cluster coherence evaluation) and that the model plateaus at 16 topics, with little interpretable improvement to be expected for more than 16 topics.

Reply: Please note that the structure of the 3 studies, as indicated above, has now changed, and the three studies are 3 phases of one big study. This should improve the interpretability of the results.

*4. Categorizing language as metaphorical or not is tricky. There is a fair amount of work on this and the most common approach uses expert coders (see, e.g., Steen et al, 2010, A Method for Linguistic Metaphor Identification). I think the approach taken in the paper is reasonable but I think some limitations should be acknowledged. For example, there would probably be some disagreement by coders over which conceptual metaphors individual instances of “fight” appeal to (war vs. boxing vs. games, etc) and even whether or not particular instances are metaphorical or not.*

Reply: Thank you. The MIP (and MIPVU) approaches for metaphor identification in texts (Steen et al. 2010) works around basically the distinction between contextual meanings and basic meanings. If there is a cross-domain comparison between contextual and basic meanings in a given text, in relation to a specific lexical unit, then that specific unit shall be marked as MRW (metaphor related word). The procedure is performed by manual annotations, using dictionaries as tools to check the contextual and basic meanings. Because the dictionaries are the same, it is (rightly) argued that different annotators shall converge to similar annotations of MRWs, and hence the procedure is reliable. In our case, we took a different approach. Firstly, our corpus is much larger than any corpus ever analyzed with the manual annotation MIP. Secondly, there are probably no good dictionaries that list among the contextual meanings of a word, the covid-related uses. It could, however, be argued that we kept somehow the distinction between contextual and basic meanings, in the following way: by selecting tweets that contain the hashtag COVID we set the contextual meanings of the words in the tweet as the pandemic-related domain. Thus, in that domain, any word that has a basic meaning related to WAR, such as all the words used in our lists, retrieved from Lakoff’s database and the web-service related-words (both solidly theoretically motivated) are used metaphorically in the context of covid.

*5. Include some discussion about the relationship between the STORM and TSUNAMI categories. At first glance it seems like all instances of TSUNAMI should also be instances of STORM, but the current approach establishes these categories as mostly (completely?) non-overlapping.*

Reply: Thank you. The overlap between the two wordlists is about 20%. It should be noted that the two frames STORM and TSUNAMI, relate to quite different concepts. On the Macmillian dictionary: STORM: an occasion when a lot of rain falls very quickly, often with very strong winds or thunder and lightning. TSUNAMI: a very large wave or series of waves caused when something such as an earthquake moves a large quantity of water in the sea. It makes sense that, denoting different natural phenomena, the two frames have quite different sets of related words.

Reply: In general, while deciding for alternative frames, and related word lists, we discarded for example the potential alternative frame FLOOD precisely because it had too much overlap with these two frames, STORM and TSUNAMI. Conversely, STORM and TSUNAMI are substantially different, and therefore both good candidates for alternative frames. Moreover, both these frames, STORM and TSUNAMI, have been indicated as prolific frames that are actually used in the discourse on Covid, by the Reframe Covid initiative (#ReframeCovid: <https://docs.google.com/spreadsheets/d/1TZqICUdE2CvKqZrN67LcmKspY51Kug7aU8oGvK5WEbA/edit#gid=268174477>). This has been now added to the description of the methods related to the Alternative Frames analyses.

*6. I like the comparison of the metaphorical frames to the FAMILY non-metaphorical frame, but the FAMILY frame also seems fairly different in that it is more of a topic than a frame (i.e. in some ways more akin to the topics identified in Study 1). I don’t think this needs to be changed, but it seems worthy of a little discussion.*

Reply: Thank you. In traditional framing theory, framing is defined as “*select[ing] some aspects of a perceived reality and mak[ing] them more salient in a communicating text, in such a way as to promote a particular problem definition, causal interpretation, moral evaluation, and/or treatment recommendation for the item described*” (Entman, 1993,p. 53).

Reply: FAMILY is a frame that, as another reviewer pointed out, can be used to talk about covid because it is an aspect of this reality. It can be used figuratively, as well as literally. Another reviewer mentioned: “*family terms COULD be figurative (and indeed, Lakoff, for example, has written much about the figurative uses of FAMILY in describing governments*” (for example, within the EU, one could say that Germany is the responsible father of the family, who scolded Italy for her behavior etc). We have chosen FAMILY deliberately to be a frame that is most likely *not* used figuratively, yet has comparable properties to a frame we expect to be used figuratively (e.g. WAR, STORM or MONSTER). In fact, we have checked qualitatively the tweets that feature one of the FAMILY terms in it, and it appears that they are used literally, not metaphorically. So, for the covid reality, FAMILY is used typically as a literal frame, which carves an aspect of the overall covid reality (the aspect related to family relations and family dynamics and family members).

Reply: Moreover, in communication sciences a frame is typically defined as consisting of two elements (Joris,d’Haenens, & Van Gorp, 2014, p. 609): framing devices which are elements in a text and specific linguistic structures (for example a list of words related to a frame) and reasoning devices which are the (latent) information in a text through which the problem, cause, evaluation, and/or treatment is implied (a more conceptual-communicative dimension of a text). A topic operationalized by topic modelling, corresponds to the first of these two components of a frame (i.e., a list of semantically related words). For example, one can say that *sister*, *father*, *home*, *parenthood*, *brotherly love* are all words that together represent the topic of FAMILY, which can be used as a frame in covid discourse: by talking about family aspects one could direct the readers’ attention to the importance of family relations and stress how these may have been disrupted by the pandemic. But a topic can also be used as a frame, if it has the communicative purpose to highlight a specific aspect of a situation to fulfil a specific communicative purpose.

Reply: We have added the definition of framing in the theoretical background, explaining that the FAMILY frame is used literally in this type of discourse (end of Theoretical Background section). We also added a paragraph on the relation between frames and topics (as intended in topic modelling) in the beginning of the section “Identifying topics in Covid-19 discourse on Twitter through Topic Modeling”.

*7. Include a note on how comparisons will be made. No inferential statistics are presented, which is common in cognitive linguistics, but the question about whether the number of cases is meaningfully different by frame or topic type will likely arise for readers.*

Reply: Thank you. We have now reported the results of a Cochran’s Q test to assess if there are any significant differences between the 5 frames, in the ways they are represented in the corpus of tweets. To do so, we compiled a tweets by frames matrix that displays binary values for presence or absence of a frame-related term in each tweet. Cochran’s Q test statistic = 47,226.72 , df = 4, p < 0.001. The post-hoc pairwise McNemar test is highly significant (p < 0.001) for all permutations (all frame pairs). We can therefore conclude that there are significant differences in the presence of target words between each pair of frames.

Reply: Additionally, we have followed a suggestion by Reviewer #4 and validated our results through a series of replication studies: we have repeated this analysis on a new corpus constructed using another two weeks of tweets; on the overall corpus of tweets encompassing 8 weeks worth of tweets, and on an external, already existing resource containing Twitter data. The results of the replication studies are reported in the section “Replication studies”. Results show that the findings are consistent across the various datasets.

*Results
1. It’s hard to interpret the results of Study 1. Are these topics the ones we would expect? Do they inform theory? Do they inform practice? Could they have come out any other way?*

Reply: Thank you. As a matter of fact, we have now restructured the paper so that these three studies are three parts of the same study, which is discussed at the end in terms of their relevance for metaphor studies and for communication sciences in general, about how pandemics are understood and discussed by the general public.

*2. What inferences can we draw about the relationship between WAR metaphors and topics from the results of Study 2?*

Reply: Thank you. Please note that the paper has been restructured. The inferences about the relationship between war metaphors and topics are already elaborated in the section LDA Topic prediction of WAR tweets and discussed in the WAR framing discussion section, from which we report hereby an excerpt: “In relation to the topic modelling of the war-related tweets, we showed that tweets that feature war-related terms are most likely to belong to topics IV, I and III, rather than to topic II. Interestingly, topic IV addresses aspects related to the reactions to the epidemics, including the measures proposed by the governments and taken by the people, such as self-isolating, staying at home, protecting our bodies and so forth. Our analysis therefore suggests that using war-related words is a communicative phenomenon that we use to express aspects of the Covid-19 epidemic related to the measures needed to oppose (fight!) the virus. Moreover, tweets that feature war-related words are also often classified within the topics I and III, which include the aspects related to communications and reports about the virus, and politics. We interpret these results arguing that public communications and political messages are likely to frame the discourse in the WAR framing. Finally, it might not come as a surprise the fact that topic II, which encompasses aspects of the discourse related to the familiar sphere, the community and the social compassion, does not relate well with the tweets containing war terms.”

Reply: Moreover, as mentioned in reply to a previous comment, we have now clarified the definitions and distinctions between frame and topic.

*3. If space is an issue, I think the comparison of the 30 vs 50 terms approaches on pp 26-28 could be cut.*

Reply: Thank you.

*4. Small point: There is no General Discussion section, although it is alluded to on p. 24.*

Reply: Thank you. We apologize. We changed the last section (which was General Discussion and Conclusions) into simply Conclusions because of space reasons and forgot to update this sentence in the text. Thank you for pointing this out. We corrected it.

*5. Small point: Include a citation (and, ideally, a more precise statistic) in the sentence “…as previous literature shows that metaphor-related words cover only a percentage of the discourse, and that literal language is still prevalent” (p. 28). What percentage? Are there other metaphors that might be prevalent?*

Reply: Thank you. We clarified this point and related it to corpus-based literature that supports this claim. In particular, we refer to the analysis performed by Steen and colleagues, who report that literal language covers 86.4% of the lexical units, while metaphor-related words cover just 13.6% of the lexical units. Their analysis is based on a corpus of 187,570 lexical units, manually annotated one by one for metaphoricity in a formal content analysis.

*6. It would be nice to ground some of the qualitative observations noted in the discussion. For example: “Words in the STORM and TSUNAMI frames seem to relate to events and actions associated with the arrival and spreading of the pandemic…” (p. 28).*

Reply: Thank you. We added examples to ground our arguments.

*7. The paper ends by introducing the idea of a Metaphor Menu, which is interesting but it doesn’t logically fall out of the current study in my opinion. Maybe this idea could be discussed a little more.*

Reply: Thank you. As we explained with our analysis, the WAR frame appears to be used in relation to specific topics, and thus to frame specific aspects of the discourse around Covid. Metaphors related to war, for example, do not appear to be apt, and therefore used, to frame the covid topics “ such as the need to feel our family close to us, while respecting the social distancing measures, or the collaborative efforts that we should undertake in order to #flattenthecurve, that is, diluting the spreading of the virus over a longer period of time, so that hospitals’ ICU departments can work efficiently without getting saturated by incoming patients.” ( excerpt taken from our Conclusions). Thus, it follows that there are aspects involved in the discourse around covid that may benefit from different figurative frames. In this sense, the war frame and war metaphors alone appear to be not sufficient to discuss all the aspects related to covid. The idea of a metaphor menu, in our argumentation, evolves from this observation, to provide communicative tools that can be used to discuss also the other topics involved in the covid discourse, where war metaphors are not observed.

____________________________________________________________
**Reviewer #2**

*The authors examined the (metaphorical) content of tens of thousands of English tweets surrounding the Covid-19 pandemic, scraped from two recent weeks from largely American twitter users. Topic modeling revealed several common themes (4 and also 16; more on this below), and that war metaphors were somewhat common (~4% prevalence), for some topics more than others, and appeared more frequently than other metaphorical domains. They argue that this is consistent with other empirical and theoretical research on the use of war metaphors in public discourse, but they now provide evidence this extends to everyday lay discourse online.
In general, I thought this was a timely article dealing with an important topic of interest to a variety of scholars, and a nice extension of previous work and theoretical musings on the use and prevalence of war metaphors. I think the methods and analyses were thoughtful and for the most part sound (though, as I detail below, I was confused about some of the details), and the results were solid. That said, I have a variety of comments and concerns that I think the authors should address in a revision before the manuscript is considered for publication.*

Reply: Thank you. *One overarching concern is that the paper feels like it was rushed to submission and therefore the writing and overall organization are not quite up to the standards of a publishable manuscript. I understand the authors’ sense of urgency in getting this paper out there while the global pandemic crisis is still at its peak, and that they literally wrote it over the past few weeks, but I think extra care needs to be taken during any revision process to make sure the writing is improved. There were many grammatical and punctuation errors throughout the paper, along with confusing sentences and shifts in tense (if they want to refer to “now,” they should stick with phrasing like “at the time of writing,” which they were not consistent with throughout the paper). At times it was difficult to follow the logic of their thinking or make sense of some of the details of the methods and analyses.*

Reply: Thank you. We have now worked on the revised manuscript to improve its readability in various ways: 1) we have restructured the sections (see comment below), 2) we have revised the tenses and 3) we have proofread the manuscript

*One of the issues is mostly organizational: the authors chose to frame their work as three “studies,” presenting the “methods” for each first, followed by the results for each, etc. I found this structure to be confusing and hard to follow, as I had to jump back and forth between methods and results and discussion sections to remember what was done (and why) as I proceeded. At one point they discuss the topics modeling results, for example, but it had been so long since they had discussed the methods, and then they waited until the subsequent section to actually give the topics meaningful labels (which they never do in the main text for the 16 topics). It was very hard to keep track of everything because of this structure.
As the research itself really strikes me as one single study, not three, but with many analytic components, I think the authors should restructure the paper in a more logical, linear fashion. For example, they could still preview the whole set of big questions and their approach in the introduction, and then the main sections could be each question in turn, with meaningful headings/subheadings rather than traditional “Study 1” and “methods” headings.
So, they could still start by describing the procedures for gathering the data from twitter and the organization of the dataset. A sub-heading in that section could be something like “Themes in the data: Topic modeling” where they go through all of the methods, results, AND discussion and labels (each with their own subheadings…) for the topic modeling. Then they can move on to a section about defining their WAR (AND alternative!) dictionaries and analyses and discussion, and then conclude with their general discussion. I think something like this would help make the flow of the paper clearer and more effective.*

Reply: Thank you. The organization of the empirical part of our paper was presented as follows:

Methods

General design of the 3 studies

Constructing the corpus of Covid-19 tweets

Study 1: Identifying topics in Covid-19 discourse on Twitter through Topic Modeling

Study 2: Determining lexical units associated with the WAR frame

Study 3: Search method for alternative framings and relevant lexical units therein

The literal frame of FAMILY used as control

Results and Discussions

General Corpus Analytics

Study 1: Topic Model Analysis

Analysis of 4 Topics

Analysis of 16 Topics

Study 1: Topic Modelling Discussion

Study 2: WAR Framing Results

LDA Topic prediction of WAR tweets

Study 2: WAR Framing Discussion

Study 3: Alternative Framing Results

Study 3: Alternative Framing Discussion

Conclusion.

Reply: Indeed, we provided in a clear and structured way the overall set of methods, and then the section with all results and discussions. The reviewer asks to restructure the paper as one big study with several analytic components. Following the reviewer’s suggestions we have restructured the paper as follows:

Experimental design

Constructing the corpus of Covid-19 tweets

General Corpus Analytics

What type of topics are discussed on Twitter, in relation to Covid-19?

Identifying topics in Covid-19 discourse on Twitter through Topic Modeling

Topic Model Analysis

Discussion

To what extent is the WAR figurative frame used to talk about Covid-19 on Twitter?

Determining lexical units associated with the WAR frame

WAR Framing Results

LDA Topic prediction of WAR tweets

Discussion

Are there alternative figurative frames used to talk about Covid-19 on Twitter?

Search method for alternative framings and relevant lexical units therein

The literal frame of FAMILY used as control

Alternative Framing Results

Replication studies

Discussion

General Discussion and Conclusion.

*Some additional comments:
While the authors reviewed a good amount of research on war metaphors, they neglected to discuss any of the dozens of articles have been written very recently about the war metaphor framing for Covid-19 (and its plusses and minuses), in both mainstream and independent outlets online. I think citing and discussing at least some of these would help situate the article in the present moment, provide additional context, and highlight the importance of the present research. Here are some examples:*

[*https://grist.org/climate/no-more-war-on-coronavirus-in-search-of-better-ways-to-talk-about-a-pandemic/*](https://grist.org/climate/no-more-war-on-coronavirus-in-search-of-better-ways-to-talk-about-a-pandemic/)
[*https://www.vox.com/culture/2020/4/15/21193679/coronavirus-pandemic-war-metaphor-ecology-microbiome*](https://www.vox.com/culture/2020/4/15/21193679/coronavirus-pandemic-war-metaphor-ecology-microbiome)
[*https://time.com/5821430/history-war-language/*](https://time.com/5821430/history-war-language/)
[*https://www.theguardian.com/commentisfree/2020/mar/21/donald-trump-boris-johnson-coronavirus*](https://www.theguardian.com/commentisfree/2020/mar/21/donald-trump-boris-johnson-coronavirus)
[*https://medium.com/@steve.howe_63053/were-at-war-the-language-of-covid-19-e3d4f4a1ae2e*](https://medium.com/@steve.howe_63053/were-at-war-the-language-of-covid-19-e3d4f4a1ae2e)
[*https://www.counterpunch.org/2020/04/24/trump-is-not-a-wartime-president-and-covid-19-is-not-a-war/*](https://www.counterpunch.org/2020/04/24/trump-is-not-a-wartime-president-and-covid-19-is-not-a-war/)
[*https://www.afsc.org/blogs/news-and-commentary/how-to-talk-about-covid-19-pandemic*](https://www.afsc.org/blogs/news-and-commentary/how-to-talk-about-covid-19-pandemic)
[*https://blogs.scientificamerican.com/observations/military-metaphors-distort-the-reality-of-covid-19/*](https://blogs.scientificamerican.com/observations/military-metaphors-distort-the-reality-of-covid-19/)
[*https://theconversation.com/war-metaphors-used-for-covid-19-are-compelling-but-also-dangerous-135406*](https://theconversation.com/war-metaphors-used-for-covid-19-are-compelling-but-also-dangerous-135406)

Reply: Thank you. All these blog posts and articles are non-academic, addressed to the large public, and most of them have been published online after our submission to PlosOne. These are the reasons why they were not included in the discussion. In fact, there has been an exponential growth of non-academic posts and articles for the large public on the language of covid, especially in relation to the recent #ReframeCovid initiative, which however gained momentum after we submitted the current paper. We gladly related to some of these more recent press materials in our academic paper (although we have already mentioned the rise of the ReframeCovid initiative), for the purpose of framing our article within the current debate, as the reviewer mentions. We added a long paragraph on this matter toward the end of the Theoretical Background section.

*On Page 8, 167, the authors say “As explained in [1], war metaphors are pervasive in public discourse and span a wide range of topics because they provide a very effective structural framework for communicating and thinking about abstract and complex topics, notably BECAUSE of the emotional valence that these metaphors can convey” (emphasis added). This makes it sound like the emotional valence of WAR is part of its structural framework, but I think this is a bit confused. War provides both a structural schema as a source domain AND it conveys an emotional tone; these points are actually separated in the paper referenced in the sentence. The authors break this down on the following page, but this sentence was unclear. Again, this may be part of the broader need to edit and revise some of the language in the article.*

Reply: Thank you, we rephrased this sentence as the reviewer suggested (as well as others throughout the manuscript, to improve readability).

*P12, Line 243-4: “…and the targeted language [English] corresponds to the first language of many (if not most) US residents.” Look this up and cite a source instead of speculating.*

Reply: Thank you. Corrected. We cited the American Community Survey (ACS), which reports this information.

*P11-12. I was terribly confused by the whole data gathering and filtering procedure. It was unclear how many tweets there were vs. individual tweeters vs. used tweets. The table tracks cumulative tweets but didn’t say that, which was confusing. It was not explained how the filtering was done (i.e., how did you choose which tweet to keep from each user that posted multiple tweets? Did the same tweeters post on multiple days and how was that dealt with?). I think this whole section could be streamlined and made much clearer.*

Reply: Thank you. We have edited the paragraph including an additional discussion about the individual tweeter filtering and improved the description of the table. We have simplified the interpretation by explaining that all 203,756 tweets are from individual tweeters, as each day tweets are filtered against the list of previous tweeters.

*Lines 270-72: The authors note they expected to find broader and more generic topics when they included 4 as compared to 16 topics. Well, of course, how else could that have turned out? In general, I found the use of two sets of topics to be unnecessarily confusing and did not feel it added much to the overall message in the paper. I suggest the authors stick with one set of topics that have easily identifiable and meaningful labels/clusters of attributes. Perhaps they could split the difference and choose 8 or 10 topics. Whatever makes the most sense for interpreting the metaphor data later is fine. I should also note this was all very exploratory/arbitrary, which is OK, but perhaps should be noted in the text (they could add a footnote explaining that using different numbers of topics doesn’t fundamentally change the pattern of findings).*

Reply: Thank you. We looked at a more compressed analysis that includes just 4 topics, and at a fine-grained analysis that encompasses 16 topics and we were interested in observing what type of information is captured by these topics. The choice for these two numbers specifically is motivated by theoretical as well as technical reasons that we have now explained (as requested by reviewer #4). Considering the topic modelling as a clustering task, the 4 ways and 16 ways solutions appear to be coherent cluster solutions. From a theoretical perspective, we were interested in exploring whether the more fine-grained solution (which delivers many smaller and more internally coherent clusters) provided intra-class distinctions and semantic specifications that could not emerge when looking at the condensed solution (which delivers a few large and less internally coherent clusters). In fact, we provided some qualitative observations in this respect, in the discussion of these analyses. It should be noted that labelling topics in LDA is common practice, observed for example in Lazard et al, and in Tran and Lee, and in Miller and colleagues (all these works are reviewed in the Theoretical Background section of our paper). Finally, again in the section Discussion of the Topic modelling analysis, we provide a discussion of the 16-way topic analysis and describe which of these appear to be related to the use of the WAR figurative frame. Therefore, and in line with the feedback received from the other reviewers, we are going to keep both the 4-way and the 16-way analyses.

*Lines 309-310: The authors write, “The term list includes the following 79 terms“… but no list was forthcoming yet until the authors discussed their other method for generating terms. Either separate out into two lists (79 + 12) or, better, use one list but BOLD the ones coming from tool two (metaNet), and do not say “the following terms…” until you are actually planning to list the terms.*

Reply: Thank you. We fixed this wording. However, we have not bolded the terms coming from metaNet or separated the two lists because it did not add value to our argumentation, and because some of the words appeared in both resources and therefore attributing them only to the first resource would have been misleading.

*The authors use FAMILY as their “literal” comparison, but it should be noted that family terms COULD be figurative (and indeed, Lakoff, for example, has written much about the figurative uses of FAMILY in describing governments…). For example, “all Americans are one family.” “the president is the father of the American household,” etc. Is there any way to check to make sure all of the instances of family terms in the dataset are indeed literal and not figurative (and to remove the latter)?*

Reply: Thank you. We manually checked the data in our corpus and indeed, as we expected, here the FAMILY related words that are used to construct the family frame are used in their literal sense. It makes sense because people on Twitter mention their personal situations in relation to Covid-19, which involves their family members, family-dynamics affected by the pandemic and the lock down and so on. So, family-related words are used literally. We have also mentioned that indeed this frame can be used metaphorically in other contexts (e.g., to talk about nations and governments, as Lakoff extensively demonstrated), at the end of the Theoretical Background section.

*On lines 503-4, the authors note that war words have a “very negative valence, OF COURSE” [emphasis added]. But I am not so sure I agree with that. Some people might get excited and motivated by ‘FIGHTING” the virus (which feels much less negatively valenced than THREAT, for example). Especially in the United States, which comprises many subcultures that glorify guns and wars and the military, I think some of these terms may be quite positively valenced. Maybe draw on some empirical work and use actual ratings of emotional valence of these words (e.g., using Pennebaker’s LIWC or some other database)*

Reply: We are grateful for the remark, nonetheless we can find our claim confirmed using emotional valence ratings. Analyzing our WAR term list using Pennebaker’s LIWC (for Social Media, including Twitter) results in a score of **28.9** for negative emotions on our terms, where the average texts have a score of **2.10** (the higher the score, the more negative the emotions). Our terms have a positive emotion score of **1.1**, where the average texts have a score of **4.57**. This indicates that war words have indeed a very negative valence.

____________________________________________________________
**Reviewer #3**

*This paper adopts a topic modelling approach to study a dataset consisting of just over 200,000 tweets about Covid-19 posted in English (and primarily from the USA) in March and April 2020. The approach is employed to: identify the main topics in the data (set at 4 and 16); study the prevalence of a WAR metaphorical framing; compare that framing with three alternative metaphorical framings and a literal topic; and investigate any correlations between the WAR framing and the topics that were automatically identified. The findings are relevant, if somewhat predictable: the WAR framing is more prevalent than the alternative metaphorical framings, and it tends to correlate with discussions of diagnosis and treatment.*

Reply: Thank you. Although the reviewer mentions that the results may appear to be somewhat predictable, there was no quantitative empirical evidence published in support of this intuition. Therefore, our paper provides empirical support for this intuition (in addition to several innovative aspects, such as the relation between the WAR related terms and specific topics of this pandemic).

*Concerning the creation of the dataset, the authors provide some justification for limiting tweets from the same account to one. However, this makes it impossible to capture the actual prevalence of the various framings on Twitter. The consequences of this decision should therefore be explicitly acknowledged.*

Reply: Thank you. We limited the tweets to one tweet per user, to construct our balanced and representative corpus, precisely because we wanted to explore to what extent Twitter users use the various framings. By doing so we limited the bias of having for instance many tweets by the same user who, arguably, uses (or does not use) a specific frame because it is a frame that he/she finds apt and appropriate, or because he/she particularly likes it. For example, imagine that one specific Twitter user is very fond of sci-fi and monsters-related issues, he/she might use the MONSTER framing very frequently in their tweets, also about Covid-19. If we kept all the tweets by this user, this might have biased the distribution frequency of the MONSTER related words. Instead, by limiting one tweet per user, and dropping the retweets, we kept our corpus relatively manageable from a technical perspective (not too heavy) as well as representative and balanced from a theoretical perspective. In other words, we are not interested in the “actual prevalence” (the reviewer means the absolute prevalence?) of the various frames on Twitter, but the relative coverage of the different framings used to talk about Covid on Twitter. We have explicitly acknowledged these matters in the section “Constructing the corpus of Covid-19 tweets”.

*The labelling of the groups of terms associated with each automatically generated topic imposes more coherence on each set of words than is actually the case, especially in the version of the analysis that only involves four topics. This is typical of this kind of computational approach to discourse analysis, but it should minimally be pointed out as a methodological issue.*

Reply: Thank you. In topic modelling this is common practice. The clusters of words returned by the topic model are unlabeled, and are typically interpreted and labelled by the analysts (as in much of the literature mentioned in the Theoretical Background section: in Lazard et al, and in Tran and Lee, and in Miller and colleagues.) We have now explained, when introducing topic modelling, that this is the case, in the beginning of the section “Identifying topics in Covid-19 discourse on Twitter through Topic Modeling”.

*As for the alternative metaphorical frames, the terms under TSUNAMI are generally to do with natural disasters, rather than tsunamis specifically.*

Reply: Thank you. The word lists were constructed using two tools widely used and acknowledged in the scientific literature: the repository of metaphors and frames released by Lakoff and colleagues (hosted at Berkley) and the RelatedWords web service. We have extensively explained both in the manuscript. The specific way in which word lists were drafted is clearly and transparently explained in the manuscript, and backed up by solid scientific literature, not by personal intuitions.

*Finally, it should be acknowledged more explicitly that this kind of analysis cannot shed light on how the WAR framing, or any other framing, are actually used. For example, it cannot distinguish between cases where the WAR framing is adopted and where it is critiqued (as has also been the case on Twitter). Ideally, the subset of tweets that employ WAR-related vocabulary could have been subjected to a more fine-grained analysis, but this usually goes beyond the scope of studies such as this.*

Reply: Thank you. Indeed, as the reviewer concludes, a qualitative analysis of the subset of tweets that use the war-related words goes beyond the scope of this study. As a matter of fact, here we are talking about 9,502 tweets. We speculate that, because the WAR frame is particularly conventionalized, most of the WAR related words are used subconsciously and therefore adopted, rather than deliberately and consciously opposed and critiqued. But this intuition implies a different set of research questions, empirical analyses and hypotheses, as also the reviewer points out.
__________________________________________________________________
**Reviewer #4**

*Thanks for the opportunity to review.

Interesting look at how discussion on Twitter may be framed using frames from the disease literature, and a brief discussion of results of topics models on a limited Twitter dataset. This is certainly a timely thing, so I recommend major revisions. With work I think this could bring value to the public health community as we endeavor to perform contact-tracing and subject to mis- and dis-information around this pandemic.*

Thank you.

*---------------------------
Main critiques
---------------------------

What I am missing is the theoretical and practical contribution. Specifically, how would the authors answer the "so what" if the tweets are framed like WAR, STORM, etc., and "so what" if they're not? (which, they're not - 90-95% of the posts are not according to the results.)
- Are relative frequencies of frames statistically different from each other, and do they happen often enough to be significant in general? Put another way, does this frame analysis work or matter on Twitter?
- How would the authors characterize the other 90% of the discussion, and why / how is it important? Are there any themes related to mis- or dis-information, or to political polarization?*

Reply: Thank you. We made explicit our goal at the end of the Introduction: “This paper reports and discusses a series of corpus-based quantitative analyses on the figurative framings used in pandemic-related discourse. In particular, the WAR frame, which previous studies have identified as pervasive in many crisis-related texts, is investigated by means of automated methods (topic modelling) applied to real-world data. By answering the research questions outlined above we claim that our data can help to improve surveillance and forecasting about the disease, as previous research on pandemics has shown (see next section).”

Reply: Thank you. We have now reported the results of a Cochran’s Q test to assess if there are any significant differences between the 5 frames, in the way they are represented in the corpus of tweets. To do so, we compiled a tweets-by-frames matrix that displays binary values for presence or absence of a frame-related term in each tweet. Cochran’s Q test statistic = 47,226.72 , df=4, p < 0.001. The post-hoc pairwise McNemar test is highly significant (p < 0.001) for all permutations (all frame pairs). We can therefore conclude that there are significant differences in the presence of target words between each pair of frames.

Reply: Additionally, we have replicated our analyses on an updated corpus of tweets collected over two months with the same criteria that we used to collect the original corpus, and we replicated our analysis on a new corpus, extracted from an existing dataset “Coronavirus Tweets Dataset” (Rabindra Lamsal. (2020). Coronavirus (COVID-19) Tweets Dataset. IEEE Dataport. <http://dx.doi.org/10.21227/781w-ef42>). We could replicate similar proportions across the same timeframe in the alternative dataset and in our updated corpus. We have included these results in the section “Replication studies”. The distributions of the frames is very similar across the three resources, with the literal (control) frame being the most frequent, the WAR frame being the most frequently used frame, among the various metaphorical frames considered, followed by the STORM, the TSUNAMI and finally the MONSTER frame.

Reply: The rest of the covid tweets on Twitter are characterized by other frames, which can be literal or metaphorical. Arguably, they are mostly literal, because the WAR frame has been indicated as a pervasive metaphorical frame to talk about diseases. Our analysis is the first contribution that tests this qualitative claim, by running an analysis on an extensive corpus of tweets, about covid. This is new quantitative information, in cognitive linguistics and metaphor studies, which wasn’t available before. As we now mentioned in the paper, in the Discussion to the alternative frames section, Steen and colleagues, for example, reported that literal language covers 86.4% of the lexical units in a corpus of 187,570 lexical units analyzed by them (a subcorpus of the BNC), while metaphor-related words cover just 13.6% of the lexical units. Their analysis encompasses all parts of speech, including for example prepositions that are very commonly used metaphorically (e.g., IN, whenever referred to a non-spatial relation). Assuming that this percentage can be applied to any text (around 13% of words used metaphorically, the rest literally) our findings provide an argument for the actual pervasiveness of the WAR frame, which covers more than one third of ALL the metaphorical language used in texts.

Reply: Disinformation and political polarization are themes that are not related to the scope of this paper.

*Second, I have concerns about sampling bias. This amounts to a study of 12 days' worth of tweets, only a few thousand. Line 60 states 16k tweets are posted every hour (do the authors have a citation?), and yet the authors collected 25k tweets per day. This equation does not balance, even when accounting for a 1% sampling rate from the Twitter API. This uncertainty undermines the efficacy of this paper - either the collection has a problem or the statement is false.*

Reply: We could not collect every tweet that relates to the covid-19 discourse on every day, since those data sets that did, have collected between 0.5 and 3.5 million tweets on a single day (depending on different keywords), quickly accumulating unmanageable amounts of data exceeding the scope of our technical limits. “*16k tweets are posted every hour (do the authors have a citation?), and yet the authors collected 25k tweets per day. This equation does not balance*” should be elaborated further: We have queried 25k tweets on each day. This query collects **every** tweet with the related keywords in chronological appearance and stops when 25k of tweets have been reached. As the collection finished after about 1.5hrs, we can infer that around 16k tweets are posted every hour (relating to the covid19 pandemic). We have rewritten this section to make this clear.

Reply: Regarding the sampling bias, we can now refer to the results from our updated corpus and the alternative existing corpus (see next response). Notably, another great influence on the amount of tweets in our sample are the restrictions (only individual tweeters, etc.) which we have discussed in the manuscript and in further responses to reviewer #2. For example, we have collected 6x as many tweets from every day for the two weeks from an alternative dataset (Lamsal - see next response), yet after applying the same restrictions we ended up with a corpus of just 2x the size. This implies that our sample is naturally much smaller than the available data that consists of retweets and tweets from multiple users - tweets that are of no use for our analysis.

*Regardless, at the time of data collection multiple datasets of Twitter related to COVID-19 existed. I strongly recommend repeating this analysis in two ways to see if the results change or hold:
- one, now that the authors have been collecting more data for a while,
- and two, perhaps more pressingly, using one of the public open datasets for Twitter with millions of tweets. See e.g. this collection of resources:* [*http://www.socialmediaforpublichealth.org/covid-19/resources/*](http://www.socialmediaforpublichealth.org/covid-19/resources/) *"Twitter Data"*

Reply: We have included a paragraph in the revised manuscript that shows how our results about the impact of WAR terms hold when we look at our updated corpus (59 days of collection) of 654,354 tweets (Section Replication studies). Analyzing all tweets from the updated database, 5.54% tweets contained at least one term from the WAR framing, which previously was 5.32%. We argue that the increase of >0.22% in the WAR framing can be partially explained by new debates entering the discourse. For example, we could identify the topic of increased domestic violence (as a consequence of the lockdown), increased cyber criminality with “attacks” exploiting the anxiety during the pandemic and the increased involvement of the military in supporting and restricting the public. It will be highly interesting to separate those topics and observe them in another topic modeling analysis, yet we do not include such analysis as it would change the nature of the paper, which is focused on the early stage of the discourse.

Reply: We agree that the size of our initial dataset was limited and therefore it is crucial to replicate the results using more data. Yet, our criteria (individual tweeter, English only and the specific keywords) are very specifically tailored and embedded in our theoretical considerations. Nonetheless, we replicated our analysis on a new corpus, extracted from an existing dataset “Coronavirus Tweets Dataset” (Rabindra Lamsal. (2020). Coronavirus (COVID-19) Tweets Dataset. IEEE Dataport. <http://dx.doi.org/10.21227/781w-ef42>). A comparison of the Lamsal dataset with our dataset over the same time-frame can be seen in the Figure below. Notably, the relative order of proportions is the same as in our analysis: Family > War > Storm + Tsunami + Monster. Differences (Family proportion decreased by 3.46%, War increased by 1.62%) can be explained by different keywords that have been used to acquire the Lamsal dataset, e.g. it includes “Corona”, which is arguably a more colloquial expression that we did not use to select our corpus. Moreover, their keywords change multiple times during the process of data mining, while we kept the same set of keywords. Whereas the suggested dataset from the online repository at [*www.socialmediaforpublichealth.org/covid-19/resources/*](http://www.socialmediaforpublichealth.org/covid-19/resources/) "Twitter Data" includes all languages, which made it infeasible to apply language identification on such a large dataset.

Reply: Additionally, we have compared our updated corpus (two months of tweets) with the Lamsal corpus covering two months of tweets (also depicted in the Figure below). We can observe that the distributions of tweets within each frame follows the same trends across the various corpora: the data collected on the 2 weeks corpora (ours and Lamsal’s) are very similar (compare bars with bright colors to bars with pastel colors). This figure along with the numerical results and a brief discussion have been added to the updated manuscript, in the section Replication studies.

*(This also suggests an opportunity to do temporal analysis, to see if the frames and discussion have changed and if so, how they are changing. This may help with a practical contribution - to answer if discussions are moving in a healthy or helpful direction, or the opposite, and why?
- For example, how often do these topics found happen over time, how often do these frames happen over time, and why is that important?*

Reply: Thank you. We did not identify a change in the distribution of the figurative frames within the discourse on Covid, within our initial 14 days of observation. With our updated corpus of 2 months of tweets, we also did not see a change of the proportion with which the frames occur. The Figure below depicts the percentage of tweets containing at least one term from the respective frame over the course of 2 months (dashed lines indicate the projection over 3 missing days, due to technical issues with the Twitter API. Notably, similar issues can be found in all other publicly available datasets). A thorough statistical analysis of these observations and the correlation of this temporal analysis with the progression of government regulation, infection rates and news are beyond the scope of the current paper. But we are currently pursuing this study, for a different contribution that will have a longitudinal perspective and will show how the different topics and frames change across time, week after week.

*How would we interpret these topics, and why might they be important? How do these frames correspond with hashtags or the literal discussion of the disease?)*

Reply: Thank you. In the manuscript we have provided a description of the topics identified by LDA, which is indeed our interpretation of the topics (including their labelling).

Reply: The only hashtags that we have considered in our analysis were the hashtags used to construct the corpus of tweets (#covid etc.). We did not analyze any hashtag related to the frames, because it was not in the scope of our analysis (see our research questions).

*Thirdly, please see critiques of the methods, related to LDA and Twitter pre-processing.*

Reply: Thank you. We replied to those.

*Fourthly, I also include more minor points and notes about statistical significance.*

Reply: Thank you. We replied to those.

*---------------------------
I also have concerning methods critiques that may undermine results:
---------------------------

On tweet processing decisions:
- I'm struggling to understand why the authors eliminated all but one tweet per user. This is a limitation. It looks like the methods and results are at the level of a tweet, not at the level of a user. In addition to the sampling bias, the authors could be discarding data that is important to their analysis. If the authors insist on retaining only one tweet per user, how this was performed? Was this random? If not, this could bias one's data again.
- I'm struggling to understand why the authors excluded retweets and mentions. How many retweets and mentions are there? Together, these choices severely limit the amount of analysis possible, to show how often the frame of the discourse is spreading, occurring, or changing. I understand wanting to exclude them initially, but what about repeating the analysis with them included to see how it changes?*

Reply: Thank you. We constructed a corpus, which follows specific criteria that we motivated in the paper. A corpus is a balanced and representative collection of texts. As we explained in the corpus, we limited one tweet per user precisely to avoid having a biased corpus where super-tweeters could have biased the percentages of frequency distribution of frames. Imagine for example that one specific Twitter user who uses Twitter many times per day, is very fond of sci-fi and monsters-related issues, he/she might use the MONSTER framing very frequently in their tweets, also about Covid-19. If we kept all the tweets by this user, this might have biased the distribution frequency of the MONSTER related words: we would have observed a high percentage of tweets with this frame, but these are all produced by the same person, who simply happens to tweet very often. This is not representative for the population. Instead, by limiting one tweet per user, we kept our corpus relatively manageable from a technical perspective (not too heavy) as well as representative and balanced from a theoretical perspective. We also dropped the retweets and mentions because these are duplicated texts, so, the same datapoint would have been counted twice. We argue that this is not desirable, from a corpus linguistics perspective. We have explicitly acknowledged these matters in the section “Constructing the corpus of Covid-19 tweets”. To conclude, our choices for the configuration of the corpus are theoretically motivated and clearly explained in the paper. We do not expect the reviewer to necessarily like them, but methodologically speaking, they are motivated and transparent. As explained later, we have added further analyses to replicate our results on an updated corpus constructed with the same criteria, collected by ourselves, and on a corpus constructed on the basis of existing resources (section “Replication studies").

*On LDA implementation:
- Did the authors use Gibbs sampling or variational inference? Gibbs sampling has been shown to yield vastly superior topics. I'd recommend repeating analysis if used variational inference and see if results hold.*

*- how did the authors choose 4 vs 16 topics? why not other numbers? did they check perplexity - what number of topics has the lowest perplexity? (most likely to explain the data)*

Reply: In the manuscript we have used an online variational Bayes (VB) algorithm through Gensim’s LDA-Multicore implementation. The topic numbers 4 and 16 have been chosen intentionally based on two factors. 1) We have observed the words in 4, 8 and 16 clusters and identified 4 and 16 to be semantically most informative and insightful. 2) We have compared the coherence values over 1 to 16 topics using C_v_ coherence (Syed, S., & Spruit, M., 2017). It is fair to assume that the slight increase of coherence after 16 topics is negligible given the fact that more and more topics might be more coherent, yet not more insightful or semantically meaningful. Therefore, we have chosen 16 topics at the higher end of coherence scores and for a small topic number, we have avoided choosing 2 topics (with the worst coherence score).

Reply: Additionally, we have now included a comparison of the coherence score over 1-16 topics using Gibbs sampling (through Mallet’s Gensim LDA wrapper). We are grateful for bringing this to our attention and report that our results hold. Firstly, the coherence score does not greatly change between the two algorithms (see Figure below). Secondly, we have investigated the actual topics (4 and 16) for both algorithms and although some of them are different (as to be expected), there are no greatly different topics or insights to be expected. We have included the trained LDA model for 4 topics and 16 topics using Gibbs Sampling in the online repository at Models/lda_N4_Gibbs.pkl and Models/lda_N16_Gibbs.pkl.

*- How did the authors handle hashtags and URLs and usernames? These may contain information, or not, depending on the design of the study. What happened? These may be useful to report if analyzing the discussion.*

Reply: Thank you for your questions. We now make it more explicit in the paper by stating that “[i]n our study design, we are not interested in the dynamics within the social network and have not collected retweets and did not investigate usernames, hashtags or URLs.” We have simply included usernames, hashtags and URLs in the frame analysis and LDA training data. Which is for example why we have observed @realdonaldtrump in a topic word cloud.

*On LDA interpretation and results:
- the authors look at significant words in topics, but what about tweets most about those topics? it can make a difference, per the coming citation. I recommend evaluating topics in both ways, as it may affect results of lines 585-596. See* [*https://scholar.google.com/scholar?hl=en&as_sdt=0%2C21&q=reading+tea+leaves+humans+interpret+topic&btnG=*](https://scholar.google.com/scholar?hl=en&as_sdt=0%2C21&q=reading+tea+leaves+humans+interpret+topic&btnG=)

Reply: Thank you for this reference. Yes, we have looked at most significant words in the topics (similar to *word intrusion* in the referred paper). In our section “LDA Topic prediction of WAR tweets”, we are in fact looking at those tweets most about the topics. Admittedly, we only do this for tweets that contain a word from the WAR frame and not for the topics themselves in order to validate their cohesion (similar to *topic intrusion* in the referred paper). Overall, we now include a paragraph in the revised manuscript that will be more critical about the proposed topics, highlighting the limitations and suggested improvement of the modeling approach as it stands (see Discussion of LDA Topic prediction of WAR tweets). Arguably, there are many ways in which we can adopt and improve the topic modeling, yet our interpretation and especially the analysis of the framing within those topics has been done on a word and tweet level.

*- did the authors interpret all of the 16 topics like they did the 4 topics? (lines 450-453)*

Reply: We have interpreted all of the 16 topics in the sense that we relate them to the 4 topics (lines 454-463). Here, we also interpret novel topics (line 461-463).

*- for topic figures, I suggest putting names of topics in the figure axes where possible. without them it's inconvenient to remember which is which*

Reply: Thank you. However, because the labels for these topics have been provided by us, rather than being returned by the model, we believe that indicating these labels in the figures would be misleading. In other words, the labels of the topics are our own interpretation of the results of the LDA analysis. The topic model provides numbers for the topics (1,2,3 and 4), and based on the clusters of words within each topic we provided a label.

*On Twitter pre-processing, I'm worried about the authors' use of general-language tools on tweets which have been shown to use vastly different language structure. - stopwords from 2012... check/justify that these are up-to-date and apply to Twitter? need to come up with domain-specific ones? - along the same lines there's a twitter tokenizer (e.g., stanfordNLP, NLTK) that are custom-built for this... what about emoji, how were these handled?*

Reply: Thank you. This is valuable feedback and we are grateful for pointing out these specifics. With respect to the general-language tools, we have to explain that we have used stopword-removal, lowercasing and tokenization for the pre-processing. Firstly, our stopwords list was an updated version and we did not only use the original list from 2012, we have included domain specific stop “words” such as “http”, “&amp” and new format stop words e.g. “don’t, don`t, I'm, I`m”. The manuscript is now updated to clearly state that we have used an updated stop word list.

Reply: As for the Twitter tokenizer, we were not aware of a specific Twitter tokenizer especially for the language library (Gensim) that we have built the analysis with. Due to the raised concerns, we have investigated the source code of the NLTK TweetTokenizer ([www.nltk.org/_modules/nltk/tokenize/casual.html#TweetTokenizer](https://www.nltk.org/_modules/nltk/tokenize/casual.html#TweetTokenizer)) in order to understand its possible improvements over our approach and whether or not they are [negligible](https://www.dict.cc/englisch-deutsch/negligible.html). The TweetTokenizer removes user handles (@). We are actually indifferent in our analysis about those as we have mentioned before. It removes html entities, which we partially remove with our additional stop words. Notably, it removes repeated characters (heeeeey), which we do not. Yet, we filter tokens with a length < 3 (“as”, “aa”, “to”, “yo” etc). It handles emoticons, emoji differently than we do. We agree that a Tweet tokenizer might reduce the number of tokens overall, but investigating our tokens, our pre-processing and stop words can compare with the same relevant tokens. Yet, we fully acknowledge the possible improvement for any future work.

*- line 279: better would have been to use tf-idf and leave the common terms in... these would have been reduced by the weighting organically*

Reply: Thank you. We are grateful for this advice. Irrespective of the removal of common terms, we did not see any improvement of the LDA w/ or w/o tf-idf, as mentioned in the manuscript.

*On literal framing control:
- What about the literal frame "it's a disease"? The authors chose family as a literal frame- this may strongly coincide with incidence or deaths from the disease, which may not be exactly what the authors want to measure.*

Reply: Thank you. We have considered several literal frames, including DISEASE and VIRUS. However, these words used within these frames are indeed often related to the figurative frames used for the analysis (fight, defend, attack, for example). In this sense, a frame like DISEASE or VIRUS, are hyper frames, they are at a different level of abstraction, compared to WAR, STORM, FAMILY, etc. Within the DISEASE frame we could still find the frames of FAMILY, WAR etc. We picked FAMILY because, instead, it appears to be more on the same level of abstraction of the figurative frames.

*- In addition... how are the authors controlling by including this? Should this be used for normalization, or testing statistical significance of frequencies or of differences among frame?*

Reply: Thank you. The literal frame is used as “control” in the sense that it is used to compare the frequency distributions of the figurative frames (metaphorical frames: war, storm, etc). As explained above, however, the statistical distribution of frequencies cannot be not easily calculated. We opted for a couple of replications, to show in a descriptive manner that the percentages are substantially preserved and the ranking of use of the frames too: the most frequent frame that occurs in the corpus (and in the secondo corpus collected for replication, and in the external corpus on which we ran our analysis for comparison) is FAMILY; followed by WAR (the most frequent metaphorical frame), followed by STORM, then TSUNAMI and eventually MONSTER.

*---------------------------
On Results and discussion
---------------------------

table 2 - are these results statistically significant? This would give weight to the authors' statement about the relative amount.*

Reply: Thank you. In response to Reviewer #1 (Remark #7), we have provided statistical (significant) results for the relative difference of proportions (using Cochran’s Q - a multi-sample extension of the McNemar test).

Reply: We used the Cochran’s Q test to assess if there are any significant differences between the 5 frames, in the ways they are represented in the corpus of tweets. To do so, we compiled a tweets by frames matrix that displays binary values for presence or absence of a frame-related term in each tweet. Cochran’s Q test statistic = 47,226.72 , df=4, p < 0.001. The post-hoc pairwise McNemar test is highly significant (p < 0.001) for all permutations (all frame pairs). We can therefore conclude that there are significant differences in the presence of target words between each pair of frames.

*lines 512-536, about topics predicting occurrences of frames... are these differences between frequencies statistically significant? are these frequencies high enough to matter?*

Reply: Thank you. We will clarify the statistical significance in this case: the reported frequencies are probability distributions that are being inferred by our LDA model. To the best of our knowledge it is non-trivial to provide a p-value for this prediction, because its accuracy relates to the goodness of the LDA model. The model itself provides a probability for each tweet to belong to a topic. As topics #1, #3 and #4 show a probability of >24% and topic #2 a probability <8%, we have interpreted this prediction with respect to the quality (coherence of our model) and concluded a meaningful difference. If the reviewer is aware of any statistical test that can be applied here, we would greatly appreciate the feedback and include it.

*Continuing down the path about frames vs. topics:
- How often do the family or alternative frames show up in the predicted topics? like likes 383-384 for the WAR frame.*

Reply: We cannot provide absolute numbers for the occurrence of the family or alternative frames in the predicted topics, because we can only use the LDA model to infer a probability of each tweet (from family terms / alternative framings) to belong to a topic. We have provided a few more probability distributions below to show that the predicted topics are in fact different for the different frames, yet the difference is diluted when observing the 4 topics. We have decided not to include a discussion of all possible distributions (4 frames x 2 topic sizes (4, 16) = 8 distributions), as it would not contribute (nor contradict) our hypothesis.

**Family	Storm**

**Monster	Tsunami**

*- In addition, how many topics include words in the frames? This may be an indicator if the frames are even worth studying on this domain. (see 90% number and earlier comment about frequencies)*

Reply: We think there is a conceptual misunderstanding here. A topic does not include a word or not. Each “topic” is a probability distribution of all tokens. That means that a token “trump”, “news” or “family” belongs to a topic with a certain probability. Therefore, all of the topics include words of the frames, some with high probability, some with low probability. To check the probability of a word within a frame one performs an inference of that word. Doing so for all words of a frame is what we have already done with the reply to the previous question. We can look at the probability from two sides, the topics or the frames, but the distribution is the same. *Lines 55-67 do the authors have any citations for any/all of these	statements?*

Reply: Thank you. We do not believe that the text on these lines, informally describing the general situation generated by the current pandemic, require references to scientific literature. This would only disrupt the reading flow.

__________________________________________________________________
**Reviewer #5**

*General comments:*

*This article provides some interesting first insights into what topics are discussed in relation to the Covid-19 pandemic on Twitter and what metaphor frames are prevalent. It was slightly disappointing that the research questions are purely descriptive, but maybe that is inevitable for a study that must be one of the first on the topic.*

Reply: Thank you.

*What is headed 'theory section' in fact includes both a literature review on analysing tweets on epidemics and another part on metaphor theory, but the two parts are not linked well. Also, the results are not linked back to the studies featuring in the literature review.*

Reply: Thank you. Indeed, both parts pertain to the Theoretical Background and they are clearly separated because these are the two “ingredients” that we combine in our paper: social media analyses of pandemics and metaphor studies. They are not supposed to be linked well, that is why they are separated. We have, however, explained that in discourse analysis and metaphor studies topics such as the pandemics can be analyzed in terms of figurative framing, and mentioned the relevant literature.

*The paper seems to have been written quickly and was clearly not proofread.*

Reply: Thank you. We apologize for this limitation, which we have addressed in this revision. Indeed, we were probably too eager to submit, given the timely nature of the topic.

*Specific comments:*

*•	As for the corpus, how can the authors be sure that the corpus only contains tweets by non-experts?*

Reply: Thank you. We have not argued that. What we have argued is that while in a corpus based on newspaper articles, for example, the texts have been written by professionals (journalists), on social media and therefore on Twitter, anyone with an account produces texts (the tweets).

*•	For Study 2, why did the authors decide against lemmatisation? There are good reasons not to use that process, e.g. because different forms of a lemma can express different metaphor scenarios, but the reasons should be spelled out.*

Reply: Thank you. We added this specific motivation to the description of the corpus, as the reviewer suggests. Very much appreciated the constructive nature of this comment.

*•	The #ReframeCovid also includes storm, monster and tsunami source domains, and it does not only include examples from news media.*

Reply: Thank you. Now, yes. We updated this information in the manuscript, in the section “Search method for alternative framings and relevant lexical units therein”. When we submitted the manuscript, the #ReframeCovid initiative had just been proposed as a hashtag on Twitter, and the crowdsourced collection of frames was about to start.

*•	What are possible labels for the 16 topics?*

Reply: Thank you. The 16-way solution is a highly granular solution, generated by the topic model. Because the labels are not provided by the model, these have to be deduced by the analysts in an empirical fashion, by means of intuitions, basically. This is commonly done in topic modelling, but the more clusters the model generates, the more it is hard to name with an individual label each cluster (that is, each topic) provided by the model. For this reason, we discussed and provided labels to name the 4-way topic model, but we did not feel comfortable in labelling each and every cluster within the 16-way analysis. Our labels might have been easily criticized by the reviewers. For this reason, we instead opted for showing the 16 topics returned by the analysis in the form of word clouds, we discussed the 16 topics as sub-categories of the 4 topics, showed which of these 16 topics contain war-related terms (topics 2, 7, and 10), and to what type of arguments the other topics seem to refer.

*•	Why was family chosen as a literal control frame?*

Reply: Thank you for this question, which gave us the opportunity to clarify this aspect, which was also pointed out by two other reviewers.

Reply: In traditional framing theory, framing is defined as “select[ing] some aspects of a perceived reality and mak[ing] them more salient in a communicating text, in such a way as to promote a particular problem definition, causal interpretation, moral evaluation, and/or treatment recommendation for the item described” (Entman, 1993,p. 53).

Reply: FAMILY is a frame that, as another reviewer pointed out, can be used to talk about covid (it is an aspect of this reality) that can be used figuratively, as well as literally. Another reviewer mentioned: “family terms COULD be figurative (and indeed, Lakoff, for example, has written much about the figurative uses of FAMILY in describing governments” (for example, within the EU, one could say that Germany is the responsible father of the family, who scolded Italy for her behavior etc). We have chosen FAMILY deliberately to be a frame that is most likely not used figuratively, yet has comparable properties to a frame we expect to be used figuratively (e.g. WAR, STORM or MONSTER). In fact, we have checked qualitatively the tweets that feature one of the FAMILY terms in it, and it appears that they are used literally, not metaphorically. So, for the covid reality, FAMILY is used typically as a literal frame.

Reply: Moreover, in communication sciences a frame is typically defined as consisting of two elements (Joris,d’Haenens, & Van Gorp, 2014, p. 609): framing devices which are elements in a text and specific linguistic structures (for example a list of words related to a frame) and reasoning devices which are the (latent) information in a text through which the problem, cause, evaluation, and/or treatment is implied (a more conceptual-communicative dimension of a text). A topic operationalized by topic modelling, corresponds to the first of these two components of a frame (i.e., a list of semantically related words). For example, one can say that sister, father, home, parenthood, brotherly love are all words that together represent the topic of FAMILY, which can be used as a frame in covid discourse: by talking about these family aspects of covid, one could direct the readers’ attention to the importance of staying together and helping each other, rather than looking at one another as strangers and potential risks of contagion. But a topic can also be used as a frame, if it has the communicative purpose to highlight a specific aspect of a situation to fulfil a specific communicative purpose.

Reply: Finally, one reviewer suggested to use, as an alternative frame, something like DISEASE. Infact, we have tried (before settling on FAMILY) to check for DISEASE and VIRUS as literal frames. However, the lexical units within these two frames encompass words related to the figurative frames (fight, defenses, attack, etc). We realized that these (DISEASE and VIRUS) are hyper-frames, that is, they are at a higher level of abstraction, compared to the frames WAR, STORM, or the literal FAMILY. We therefore opted for FAMILY because, we argue, is at the same level of abstraction of WAR and the rest of the figurative frames.

Reply: We have added this discussion in the revised manuscript, by introducing the definition of framing in the theoretical background, explaining that the FAMILY frame is used literally in this type of discourse (end of Theoretical Background section), and by explaining the relation between framing and topics (as intended in topic modelling) in the beginning of the section “Identifying topics in Covid-19 discourse on Twitter through Topic Modeling”.

*•	"Previous literature shows that metaphor-related words cover only a percentage of the discourse": does this refer to Steen et al. (2010)?*

Reply: Thank you. Indeed. Yes. We have added this reference and elaborated this aspect further. The edited text is hereby reported:

Reply: “previous literature shows that overall metaphor-related words cover only a percentage of the discourse, and that literal language is still prevalent (e.g., Steen et al. 2010). Steen and colleagues, for example, report that literal language covers 86.4% of the lexical units, while metaphor-related words cover just 13.6% of the lexical units. Their analysis is based on a sub-corpus of the BNC that encompasses 187,570 lexical units extracted from academic texts, conversations, fiction and news texts. All parts of speech are included in their analyses, including function words (such as prepositions and articles). Assuming that this percentage of figurative language compared to literal language use can be applied to the discourse around Covid-19, we would expect to find around 13% of the lexical units in our corpus to be used metaphorically (including pervasive metaphorical uses of function words). Many of these metaphor-related words pertain figurative frames such as the frames analyzed in our paper. In this perspective, the percentage of use of the WAR frame reported in our study suggests that this frame is particularly pervasive, because it covers more than one third of all the metaphorical language typically used in texts.”
