# Supplementary material for: Framing COVID-19: How we conceptualize and discuss the pandemic on Twitter
Source: PLoS One. 2020 Sep 30;15(9):e0240010. doi: 10.1371/journal.pone.0240010 (PMC7526906; doi:10.1371/journal.pone.0240010)
Supplement: Attachment — Submitted filename: Response to Reviewers.docx [file pone.0240010.s003.docx]

To the Editor and Reviewers,

Once again, we are very thankful to the reviewers, for taking their time to provide constructive and helpful comments to our paper, which has now significantly improved in quality. In this letter we have addressed the remaining comments and indicated what we changed in our manuscript. We have then submitted the paper to a professional proofreader and editor, native speaker of American English, to proofread the text. We hope that this iteration has cleared any remaining language issues highlighted by Reviewer 1 and 2.

As for the technical issues addressed by Reviewer 4, we have provided a lengthy response and several adaptations to our paper. We are grateful for the detailed feedback, yet we have to acknowledge that some of the criticism will remain, which we attribute not to a wrong but different methodology arising from different research areas (Corpus Linguistics vs. Social Media Monitoring). Find our detailed response below.

Kind regards,

Philipp Wicke and Marianna Bolognesi

**REVIEWER 1**

Introduction & Theoretical Background

The introductory sections do a better job of describing and situating the contribution of the current work. The paragraph that explicitly details “The innovative aspect of this paper…” (p. 5) is particularly helpful.

Reply: Thank you.

There is still an issue with the sentence I highlighted in my previous review, which now reads, “Unlike the articles on magazines and journals typically used for corpus analyses of this kind, Twitter contains messages written by journalists and other experts in mass communication: most tweets are provide by non-expert communicators” (p. 4). Maybe: “Although Twitter contains messages written by journalists and other experts in mass communication, most tweets are provided by non-expert communicators…”

Reply: Indeed, we apologize, this was a typo that we didn’t catch because of the track changes, thank you for pointing this out, we corrected it.

I still find the heading “Theoretical Background” misleading. While that section certainly does discuss relevant research that helps situate the current work, it doesn’t discuss theoretical background per se (to my eye). But if the authors feel strongly that it does, that’s ok with me.

I feel more strongly that the heading “Experimental design” should be changed because the study is not an experiment. Maybe “Study design.”

Reply: Thank you. As explained in the previous letter, we do believe that the Theoretical Background discusses literature related to the topic addressed in the paper: quantitative analyses based on Twitter data related to epidemics (paragraphs 1-4); cognitive linguistic studies of figurative framings and metaphors, including the WAR frame and metaphors that we tackle in our study (paragraphs 5-7) and alternative framings (paragraph 8). This section does contain a selected review of the literature in these two fields of research, which is functional to our argumentation. We therefore remain with this heading. Thank you for your collaboration.

Reply: We changed the heading “Experimental design” into “Study design” as suggested by the reviewer.

Methods

I appreciate the revised structure of the methods and results, which makes the paper more readable. I still find the first section difficult to interpret. I think it would be useful to say explicitly in the set up or discussion of the topic modeling that the categories will be useful for understanding how the war frame is being used.

Reply: Thank you. In the setup of the study (Study Design) we explicitly mentioned: “To address our three research questions, first we explored the range of topics addressed in the discourse on Covid-19 on Twitter using a topic modelling technique. Consequently, we explored the actual usage of the WAR frame, and in which topics related to Covid-19 is the WAR frame more frequently used.” We have now made explicit in that paragraph the fact that the identification of the topics is functional to the investigation of the distribution of the war-related terms.

It’s not clear what it means to say that, “The two numbers of clusters were chosen on an empirical basis” (p. 17). What was the empirical method / criteria? How was it evaluated? Adding the LDA coherence measure is useful but seems different from what the authors mean when they say that the “clusters were chosen on an empirical basis.”

Reply: With the term “empirical basis” we mean that these numbers were initially chosen by looking at the data and then backed up by post hoc testing. Since 2 and 3 seemed intuitively to be a too small number of clusters that would not allow much observation to emerge, we opted for multitudes of 2, e.g. 4, 8, 12, 16. Then we tested with a post hoc analysis the coherence of the various cluster solutions obtained, and based on these measures, we confirmed that 4 and 16 clusters appeared to be the best choice, given our goal to have a less granular (4 clusters) and a more granular (16 clusters) solutions. Clusters in between 4 and 16 did not provide any meaningful semantic insight, which had not been generalized by 4 clusters or specified by the 16 clusters. The post hoc coherence analysis was conducted by evaluating the coherence score calculated for each cluster solution. The cluster coherence score that we adopted is typically used in topic modelling and with the LDA algorithm, and it is called “Cv measure”. This score tells us how coherent a cluster solution (that is, a topic modelling analysis) based on a number of clusters (=topics) is. The score is based on a one-set segmentation of the most important words, a sliding window and an indirect confirmation measure. The latter uses normalized pointwise mutual information and cosine similarity (for more information on this, see: M. Röder, A. Both, and A. Hinneburg: Exploring the Space of Topic Coherence Measures. In Proceedings of the eighth International Conference on Web Search and Data Mining, 2015.)

I appreciate the discussion of the MIP and MIPVU in the response letter, but I think some of these issues should be noted in the manuscript itself. Namely, this method of categorizing and quantifying metaphor is new, different from alternatives, and has its own benefits and limitations (as does any method of coding language). To be clear, I think the method being used is interesting and worthwhile. But it likely misses some instances of metaphor and it likely counts some words as metaphorical that are being used in a literal sense. Maybe, for example, someone tweeted about how COVID was spreading on the aircraft carrier the USS Theodore Roosevelt, which is a war ship. Given that one of the main contributions of the paper is the method, I think some attention should be given to the strengths and limitations of the method. Of note, a major strength of the method is that it can handle a lot of data. This point could also be made more explicitly. [The comment below also relates to a limitation of the coding method.]

Reply: Thank you, we agree and we have now acknowledged these strengths and limitations of our method in the paper, comparing our way to identify automatically metaphor-related (frame-related) words in a very large corpus of data, to manual methods such as MIPVU, as suggested by the reviewer. We added a paragraph explaining this trade off at the end of the section “Determining lexical units associated with the WAR frame”.

I agree that the concept of a STORM is different from the concept of a TSUNAMI. However, if the same words (e.g., wave, flood, disaster) are being used to index both concepts, how do we know which concept people are drawing on? For example, the word “disaster” is the second most frequent marker of a TSUNAMI in the corpus and also the second most frequent marker of a STORM in the corpus. The word “wave” is also frequent in both. This means that some of the tweets are double counted as metaphorical in a sense, no? Do the analyses assume that the categories are mutually exclusive and exhaustive? It would be useful to report the percentage of tweets that included multiple categories of metaphor.

Reply: Thank you for this comment. The fact that there are lexical entries that appear in both domains tells us that the two domains are indeed semantically related, and therefore the same lexical entries can be used in both frames. This is semantically correct, and also, to be expected. The frames do not need to contain mutually exclusive sets of words: in fact, the more two frames are semantically close to one another, or similar to one another, the more they will share lexical entries. From an ontological perspective, the idea cannot be defended that words belong to one and only one frame. Our algorithm operates in a way that is motivated by these assumptions, and therefore as the reviewer correctly observes, can attribute a tweet to both frames, the STORM and the TSUNAMI, because the calculation is based on the frequency count of a lexical entry that may be listed in both frames, such as the word “disaster”. In this sense, each framing is measured independently from other frames, and we agree that there might be overlaps. This is a sustainable perspective in terms of its ecological validity: we cannot really distinguish whether in the mind of the tweeter there was specifically a STORM frame or a TSUNAMI frame, when they tweeted. It would be unrealistic to argue otherwise. Our analyses do not assume that the lists of lexical entries used for each figurative frame are mutually exclusive and exhaustive for that specific frame. They rather assume that the lists of lexical entries for each frame are theoretically motivated , meaningful and representative for the frame they stand for. Please note that the statistical test we applied in our analyses to compare the percentages by which the different frames are used takes into account the fact that a lexical entry may belong to more than one frame (in this sense, these categories are not mutually exclusive; the presence/absence of a lexical entry in a tweet, however, is a binary, mutually exclusive property: either the word is there, or it is not).

Reply: To clarify this further, our algorithm counted 1 hit whenever a lexical unit (a word from a list) was found in a tweet. Then, the numbers were added up within each frame. In this sense, our unit of analysis is the lexical units in the word lists, not the tweets. A tweet can be counted as many times as it has words related to a frame. For example a tweet such as “This wave of #covid is hard to fight” will count 1 hit for the TSUNAMI frame (wave), 1 hit for the STORM frame (wave) and 1 hit for the WAR frame (fight). Therefore, reporting the percentage of tweets that were counted more than once does not seem to be useful. We have however reported, in the previous and in the current version of the paper, the number of tweets that encompass more than one war-related term (N=1253).

I understand and appreciate how the cluster of words related to FAMILY are being used, as a point of comparison with the figurative frames (WAR, STORM, TSUNAMI, MONSTER). But I still think there is a qualitative difference between this category and the others. The language of WAR (STORM and TSUNAMI and MONSTER) is being used to frame different aspects of the pandemic and response. In many cases, the issues being framed metaphorically could be discussed using an alternative metaphorical frame or in a literal sense. The tweets about FAMILY, on the other hand, seem to be about FAMILY per se. That strikes me as an important difference that should be discussed in the paper.

Reply: Thank you. Indeed, these are qualitatively different frames because WAR, STORM, TSUNAMI, and MONSTER are figurative, while FAMILY is literal. The reviewer mentions: “ The language of WAR (STORM and TSUNAMI and MONSTER) is being used to frame different aspects of the pandemic and response. [...] The tweets about FAMILY, on the other hand, seem to be about FAMILY per se”. This is precisely because the first are used metaphorically (and therefore with a specific effect, generated by the metaphor used, which can be replaced by using another metaphor, or by using literal language instead). The latter is literal language, thus it is used to talk about the literal referents. We have chosen FAMILY deliberately to be a frame that is most likely not used figuratively, yet has comparable properties to a frame we expect to be used figuratively (e.g. WAR, STORM or MONSTER). In fact, we have checked qualitatively the tweets that feature one of the FAMILY terms in it, and it appears that they are used literally, not

metaphorically. So, for the covid reality, FAMILY is used typically as a literal frame. In any tweet that uses a term from FAMILY the context is still the pandemic due to the nature of the dataset.

Results

See above for questions about tweets that were categorized as relating to multiple frames.

Reply: Thank you, as explained above our analyses do not require the lexical entries to belong to mutually exclusive lists. For this reason we applied the Cochran’s Q test statistic in our analyses to compare the percentages by which the different frames are used takes into account the fact that a lexical entry may belong to more than one frame (in this sense, these categories are not mutually exclusive; the presence/absence of a lexical entry in a tweet, however, is a binary, mutually exclusive property: either the word is there, or it is not). Cochran's Q test requires that there only be a binary response (e.g. word is present/word is not present or 1/0) and that there be more than 2 groups of the same size (e.g. WAR, FAMILY, STORM, MONSTER, TSUNAMI). The binary response does not need to be mutually exclusive for the groups.

Adding the replication study strengthens the paper.

Reply: Thank you.

I recommend toning down some of the claims like, “…the percentage of use of the WAR frame reported in our study suggests that this frame is particularly pervasive, because it covers more than one third of all the metaphorical language typically used in texts” (pp. 33-34). The study restricts itself to four metaphorical frames of interest; the full range of metaphor typically used in texts is not quantified here.

Reply: Thank you, we toned down the argument and clarified it further, in relation to the percentages of metaphorical language reported by Steen and colleagues in their corpus analyses.

**REVIEWER 2**

I want to commend the authors on the impressive revisions to this paper. I think they have done just about everything they could to address all of the reviewers concerns, and I feel the paper is much stronger now and will make a nice addition to the literature. Indeed, I could see myself citing the findings in a future talk or article!

Reply: Thank you, this is great news and very much appreciated.

The only reason I am selecting "minor revision" rather than "accept" is that PLOS ONE does not copyedit accepted manuscripts, and I still feel the paper could use one more round of proofreading and editing. While the overall structure of the paper is greatly improved now, there were still grammatical errors and awkward phrases sprinkled throughout that hindered readability.

Reply: Thank you, this is very helpful. In fact, we have now had a (paid) professional proofreader, American English native speaker, proofreading this version of the manuscript.

For example, lines 75-77 contained the sentence "Unlike the articles on magazines and journals typically used for corpus analyses of this kind, Twitter contains messages written by journalists and other experts in mass communications: most tweets are provided by non-expert communicators" This is very confusing to me as it seems to be saying two different things.

Reply: This is indeed a typo due to the heavy editing and track changes. The revised statement now reads: “Although Twitter contains messages written by journalists and other experts in mass communication, most tweets are provided by non-expert communicators”.

A more mild example, but still one that should be edited for clarity, comes on lines 179-180: "The military metaphor thanks to which we frame diseases such as cancer is a very common one to be found in public discourse." And in the next sentence, the authors use the article "the" before Time Magazine, which is not necessary.

Reply: Thank you for your collaboration, we acknowledge these typos and mistakes, and we corrected these specific cases even before sending the manuscript to the proofreader. Then, the proofreader took care of the whole document. Very much appreciated.

I am not going to go into every example of confusing writing, but I do want to recommend the authors go over the paper again and update the prose as necessary to enhance clarity. At that time, I will be prepared to accept!

Reply: Thank you.

**REVIEWER 4**

Thank you to the authors for drastically improving many points about the paper. I enjoyed reading this interesting research even more this time. It is much cleaner to read and understand, including contributions, backing statistical analyses, and replication on other datasets. Other reviewers also brought up great points that I did not think of.

However, as is written, the study seems to have unacknowledged limitations and concerns that render contributions too broad about understanding general discourse on Twitter that I would recommend addressing before accepting this paper.

My concern stems from ambiguity in terms of unit of analysis that biases the representativeness of the data, which causes unacknowledged impact on results and contributions. Claims cannot be made about general discourse on Twitter, but they *can* be made about a biased yet useful subset - those tweets that originate from less-frequent tweeters.

Reply: Thank you. This comment is related to our methodological choice to select one tweet per user and drop retweets, which we motivated in the previous reply to reviewers. We are here reporting our previous reply: “We constructed a *corpus*, which follows specific criteria that we motivated in the paper. A corpus is a balanced and representative collection of texts. As we explained, we limited one tweet per user precisely to avoid having a biased corpus where super-tweeters could have biased the percentages of frequency distribution of frames. Imagine for example that one specific Twitter user who uses Twitter many times per day, is very fond of sci-fi and monsters-related issues, he/she might use the MONSTER framing very frequently in their tweets, also about Covid-19. If we kept all the tweets by this user, this might have biased the distribution frequency of the MONSTER related words: we would have observed a high percentage of tweets with this frame, but these are all produced by the same person, who simply happens to tweet very often. This is not representative for the population. Instead, by limiting one tweet per user, we kept our corpus relatively manageable from a technical perspective (not too heavy) as well as representative and balanced from a theoretical perspective. We also dropped the retweets and mentions because these are duplicated texts, so, the same datapoint would have been counted twice. We argue that this is not desirable, from a corpus linguistics perspective. We have explicitly acknowledged these matters in the section “Constructing the corpus of Covid-19 tweets”. To conclude, our choices for the configuration of the corpus are theoretically motivated and clearly explained in the paper. We do not expect the reviewer to necessarily like them, but methodologically speaking, they are motivated and transparent. As explained later, we have added further analyses to replicate our results on an updated corpus constructed with the same criteria, collected by ourselves, and on a corpus constructed on the basis of existing resources (section “Replication studies").”

Reply: The reviewer has not commented on our reply, which provides a rational motivation for our methodological choices. In this sense, the comment by the reviewer stating that our analyses are based on “a biased yet useful subset - those tweets that originate from less-frequent tweeters” is not fully correct. Our analyses are based on a corpus (and then replicated on another corpus) of tweets. Decades of published research reporting empirical studies in corpus linguistics are based on corpora that are carefully constructed, based on principles such as representativeness and balance of the texts included therein. We therefore believe that our corpus of tweets, which we constructed based on a few theoretically motivated and clearly explained criteria, is representative for the way in which Twitter users conceptualize and talk about Covid on Twitter: we constructed a corpus that represents the language that people use on Twitter, as it is common practice in corpus linguistics and cognitive linguistics, as well as in many disciplines within Language sciences. Please note that we do not only consider “tweets that originate from less-frequent tweeters” as the reviewer mentions, but we rather consider one tweet per tweeter, thus taking into account also super tweeters, by including one of their tweets in the sample.

Generally, any study that performs social monitoring needs to:
- make clear what its unit of analysis is,
- then accordingly determine what it means to be representative of these units in its sampling frame,
- gather units to analyze and analyze those same units
- acknowledge any limitations of representativeness
- and limit contributions accordingly

Reply: We are frankly unfamiliar with the term “social monitoring”. We have found a definition for “social media monitoring”, which appears to be the active monitoring of social media channels for information about a company or organization, usually tracking of various social media content in general as a way to determine the volume and sentiment of online conversation about a brand or topic. This is not what we present in our study.

Reply: The information requested by the reviewer has been provided in the paper as follows:

- units of analysis: the first study is based on topic modelling, and adopts the methods described by the LDA algorithm; the other two studies are based on the lexical entries that belong to the various frames, as clearly stated multiple times in the manuscript. We believe that with units of analysis the reviewer may refer to the words (from the various lists of frame-related words) that we counted in the tweets. To clarify this further, our algorithm counted 1 hit whenever a lexical unit (a word from a list) was found in a tweet. Then, the numbers were added up within each frame. In this sense, our unit of analysis is the lexical units in the word lists, not the tweets. A tweet can be counted as many times as it has words related to a frame. For example a tweet such as “This wave of #covid is hard to fight” will count 1 hit for the TSUNAMI frame (wave), 1 hit for the STORM frame (wave) and 1 hit for the WAR frame (fight). We then reported in the paper the total number of occurrences of the words in the tweets, as this paragraph explains: “Analyzing all tweets from the database, a total of 10,846 tweets contained at least one term from the WAR framing, which is 5.32% of all tweets. Of these, 1,253 tweets had more than one war-related term."
- representativeness: the representativeness of these units is backed up by the sources where they have been retrieved (RelatedWords and the Frames in the Berkeley database of figurative framings).
- gather units and analyze those units: that is how we proceeded, indeed!
- Acknowledge limitations: we have acknowledged the limitation of our analysis, in particular the limitation due to the assumption we made, that the lexical units used for our analysis are used always metaphorically, throughout the corpus. This was necessary, in order to perform an automated analysis. We added a paragraph explaining this trade off at the end of the section “Determining lexical units associated with the WAR frame”.
- we toned down the claims about our contribution, in the General Discussion and Conclusion section.

At minimum in this particular study,
- it should be made clear and consistent through data collection and analyses whether this particular study is analyzing use of frames by users, or use of frames in tweets, (I'm pretty sure it's tweets, right?)

Reply: We analyse the presence of lexical entries related to specific frames in a balanced corpus of tweets that is constructed by taking into account one tweet per user, to avoid the bias of super-tweeters. In this sense, we do not monitor how Twitter works per se, but how people, in our case Twitter users, tend to talk about the pandemic on Twitter. Our study relates to principles and assumptions generally made in fields such as corpus linguistics, discourse analysis and cognitive linguistics, which may be substantially different from those made in the “social media monitoring” field, suggested by the reviewer. As we explained, in line with common practice in the disciplines with which we relate, we started by constructing a balanced and representative corpus of tweets that features as many users as possible to preserve variability within the corpus (one tweet per user), that avoids the bias introduced by super tweeters (explained above), and that provides a window onto the language used on Twitter to talk about the current epidemic.

- the limitation of a bias against super-tweeters should be made more explicit, and I would accordingly recommend explicitly walking back the study's claims, research questions, and contributions, because claims of general representativeness on Twitter are not defendable.
Specifically, if it is chosen to omit the vast majority of tweets by keeping one tweet per user and omitting retweets, claims cannot be made about how the general Twitter discourse discusses and frames COVID-19 (or to put it another way, the study's data is not representative of all tweets). Claims instead can be made about how those tweets *originating from less-frequent tweeters* discuss and frame COVID-19.

Reply: We have not “chosen to omit the vast majority of tweets by keeping one tweet per user and omitting retweets”, as the reviewer argues. Rather, we have constructed a corpus of tweets that is representative and balanced for the language people use on Twitter to talk about Covid, which is the phenomenon we want to investigate. Note that *any* type of sampling would indeed “omit the vast majority of tweets”. The criteria used for sampling, in our case, have been clearly explained and motivated, in line with common practices in corpus linguistics, discourse analysis, and cognitive linguistic research.

Either that, or I would recommend at least one of:
- switching to analysis at the level of a user
- more representative sample from tweeters (as one tweet per user is usually not seen as representative of a user's content, whether they are among the top tweeters or not)

Please find details below - perhaps these can make the issue more clear if it helps.

First, the study has a bias against retweeters and sharers, in favor of original tweeters, as is stated. However, many users on Twitter retweet or share someone else's tweets - this is seen as a proxy for behavior and opinion, as usually people share things they agree with.

Reply: As previously mentioned, we constructed a corpus of tweets based on the principles of representativeness and balance, in relation to the language that people use to talk about covid on Twitter. We find the reviewer’s statement “people share things they agree with” quite interesting and possibly controversial, as people may share things they strongly disagree with, by retweeting them and adding a very negative comment on them. But, as non-experts of social media monitoring, we limit our discussion to the linguistic phenomena hereby investigated.

Therefore, more accurate counts of frames might be *including* those frames that are retweeted. At the very least, one may analyze with retweets and without to see any differences. It's fine that the study disregards retweets, but this should be acknowledged as a limitation and claims about discourse should be modified accordingly.

Reply: Indeed, we have acknowledged the fact that we dropped retweets on purpose, and motivated our choice. We have modified accordingly our claims about the discourse around Covid on Twitter, explaining that our findings relate to the corpus of tweets that we constructed, and not to Twitter as a social network, which includes retweets and information propagation that can be strongly affected by super-tweeters. A new paragraph including these limitations to our study has been added to the General Discussion and Conclusion section.

And second, to paraphrase, data is being "limited to one tweet per user because of technical limitations and to make the corpus balanced and representative". I would reject the study's claim that the resulting corpus is representative - because the study is not clear about *how* it is representative. As designed, it seems like the study is confused about the unit of analysis that it is trying to represent: the tweet or the user.

Reply: As argued above, the units of analysis are the lexical entries, that is, the frame-related words, which have been carefully constructed on the basis of existing resources. We investigated how these lexical entries are used in a corpus of tweets about Covid, which has been constructed by selecting tweets that contain specific hashtags about covid, dropping retweets because they are technically repeated texts that are not original, and thus in our opinion not much *representative* of the variability in semantic content that can be found on Twitter, and keeping one tweet per user to construct a *balanced* corpus that is not biased toward the texts tweeted by super-tweeters (or even twitter bots, which are usually super tweeters).

- If the study wishes to accurately represent tweets, then by Twitter's nature there are super-tweeters, and frequencies of frames will be biased by super-tweeters who use frames more. This is an accurate representation of the Twitter discourse.
- If the study wishes to accurately represent users, then it should state this, and tweets should be aggregated or sampled representatively per user, and and all subsequent analyses should be at the level of the user, not the level of the tweet.

However, as currently designed, the study starts at the level of a user by "retaining one tweet per user", but then moves back to analyze at the level of a tweet. This results in a bias against super-tweeters with "retaining one tweet per user". Therefore, all subsequent analysis at the level of a tweet omits the vast majority of tweets that compose Twitter discourse - exactly what it seems to want to analyze.

Reply: Thank you. This study, as mentioned above, is rooted in principles and assumptions that are usually starting points in corpus linguistics and cognitive linguistics. It is physically impossible to mine the whole Twitter as it stands, because it is a dynamic archive of texts that increases on a daily basis, and therefore there are no claims that can be true for “Twitter” as a whole, technically speaking. So, we have to sample this archive, and construct a corpus that works well for our purposes. In our case we constructed a corpus that is balanced and representative for the aim of the study. Our study is to mine the linguistic phenomenon of figurative framing, that is: the use of words related to specific frames, which suggests a specific conceptualization related to the topic of Covid. Given this aim, we constructed a corpus that is suitable for this type of linguistic investigation, and that would not distort the statistic of our findings, related to the phenomena investigated.

Reply: Our choice to select one tweet per user does not go in the direction of a user-based analysis. Rather, it is done in order to have a selection of tweets that have been produced by as many different human minds as possible, rather than by a very limited number of minds (supertweeters) which may in fact very likely encompass artificial minds (twitter bots).

Reply: Regarding our methodological choice to remove duplicates (retweets), please note that even in the construction of corpora based on the texts found in the web (e.g., UK WAC, IT WAC etc) duplicate texts are typically dropped, to favor the representativeness of the texts included in the corpus. Our choice for the construction of the corpus of tweets goes in the same direction of these previous studies. In fact, there are several tools implemented precisely to remove duplicates texts for the construction of web-based corpora. Twitter as well, is a web-based resource, where creating duplicates is extremely easy (one click: retweet!), but the motivations behind this operation may be different (notably, agreement vs disagreement with the content of the original tweet) and hard to interpret in an automated manner.

- Minor point: The study should address exactly how it "retained only one tweet per user". This was asked in the review. Was the earliest tweet retained? the most recent? the one with the most framing words? Is it simple random sampling? I ask to elucidate, not to be flippant, as ambiguity does not lead to reproducibility - it leads to concern. A brief mention would suffice.

Reply: Thank you, we added the information that only the first contribution of each user was retained, meaning we have kept the first tweet on that day by users whose tweets we have not collected yet. In the previous response, we have explained that “The algorithm picked the first tweet per user that it could find, within the timeframe indicated.” To respond directly to the questions of the reviewer: Was the earliest tweet retained? Yes, the earliest on that given day. The most recent? No. The one with the most framing words? No. Is it simple random sampling? No, since we stated in the previous response that “[c]ollecting all tweets from all users, and then randomly selecting one per each user would have been computationally cumbersome and would have exceeded our database capacity.”

I can recommend four courses of action, although the authors would know which would be more appropriate for their desired contributions:
1. Keep current data collection, methods, and results that seem to analyze at the level of a tweet, and acknowledge explicit bias against super-tweeters, to favor those users who tweet less often. This seems to require revisions to contributions and research questions, as the overall Twitter discourse is not being studied for framing, but the Twitter discourse being studied is biased towards discourse by less-frequent users. Is there a theoretical motivation for studying in this way?
2. Keep current data collection, analyze at the level of a user, and acknowledge explicit bias against super-tweeters, to favor those users who tweet less often. This would require more extensive revisions to analyses, but would result in a different contribution: instead of studying the entirety of framing discourse on Twitter, studying how *users*, with emphasis on less-frequent users, frame the discussion.
3. Decreasing bias against super-tweeters by gathering a representative amount of tweets per user, and analyze at the level of a user. This would result in a proper representative analysis of discourse on Twitter at the level of the user, and contributions on how users discuss and frame would follow.
4. Decreasing bias against super-tweeters by gathering a representative amount of tweets per user, and analyze at the level of a tweet. This would result in a proper representative analysis of discourse on Twitter at the level of the tweet, and defendable contributions on how tweets generally discuss and frame would follow.

Reply: Thank you, we adopted the first course of action and acknowledged the limitations of our study accordingly, emphasizing our background in cognitive and corpus linguistics and where our methodological choices stem from.
