# Supplementary material for: Framing COVID-19: How we conceptualize and discuss the pandemic on Twitter
Source: PLoS One. 2020 Sep 30;15(9):e0240010. doi: 10.1371/journal.pone.0240010 (PMC7526906; doi:10.1371/journal.pone.0240010)
Supplement: Attachment — Submitted filename: Response to Reviewers.docx [file pone.0240010.s004.docx]

Dear Dr Panos Athanasopoulos, Academic Editor at PlosOne,

Please find enclosed the third revised version of our manuscript Framing COVID-19 How we conceptualize and discuss the pandemic on Twitter, PONE-D-20-10986.

Once again we are very thankful to the reviewers, for taking their time to provide constructive and helpful comments to our paper, which has now significantly improved in quality. In this letter we have addressed the remaining comments and indicated what we changed in our manuscript.

As indicated, we are hereby enclosing:

•	A rebuttal letter that responds to each point raised by the academic editor and	reviewer(s), labeled 'Reply to Reviewers'.

•	A marked-up copy of your manuscript that highlights changes made to the original	version, labeled 'Revised Manuscript with Track Changes'.

•	An unmarked version of your revised paper without tracked changes, labeled	'Manuscript'.

We look forward to hearing back from you in due course.

Kind regards,

Mr Phil Wicke

Dr Marianna Bolognesi

**REVIEWER 1**

The manuscript, which explores metaphors used to frame COVID-19 on Twitter, has been revised to address concerns raised in review. Overall, I think the revision is very successful. The argument is much clearer, and I think the paper is nearly ready for publication. I have a few final comments that should be straightforward to address. I am recommending that the paper be accepted.

Thank you. We appreciate the constructive feedback throughout the review process and such successful revision would not have been possible without such useful and detailed reviews.

1. I still think the use of “empirical basis” is misleading and confusing (p. 17) because, in many research contexts, this term is used to refer to specific criteria or quantitative methods of comparison. Since the current approach is more informal, why not say in the paper what is said in the response letter? Namely, something like, “These two numbers of clusters chosen by looking at the data and are backed up by post hoc testing.”

Thank you. We have now changed the use of “empirical basis” and refer to the selection of clusters as mentioned by the reviewer.

2. Please include some details about the criteria that Lamsal used to create their database of COVID-19-related Tweets.

Thank you. We have now included the following criteria in the paper: Lamsal’s dataset is a constantly updated repository of twitter IDs. The collection of those IDs is based on English tweets that include 90+ hashtags and keywords that are commonly used while referencing the pandemic.

3. I was a little confused by the term “individual” in “individual tweeters” on page 13. I think “unique tweeters” would be clearer, assuming that is the intended meaning of “individual tweeters.”

Thank you. We have now changed the mention of “individual tweeters” with “unique tweeters”.

4. Using the Topic labels (e.g., “Communications and Reporting”), rather than the numeric codes (e.g., “Topic III”), would make the figures more readable.

Thank you. We have now updated the figure with the topic labels for the 4 topics as requested by the reviewer.

Once again, I found this research really interesting and think that it will make a nice contribution to the literature. I appreciate all the careful work the authors have done throughout the review process.

Thank you, we appreciate this comment and all the careful responses from the reviewer.

**REVIEWER 2**

I am satisfied the authors have addressed all critical reviewer points. The current manuscript is significantly stronger than the initial submission and makes a nice contribution to the literature.

Thank you. We appreciate the constructive feedback throughout the review process and such successful revision would not have been possible without such useful and detailed reviews.
